# Experiences of the International In-Training Examination (I-ITE) by Rwandan pediatric residents – a survey-study describing candidate feedback

**DOI:** 10.12688/f1000research.27293.2

**Published:** 2023-01-06

**Authors:** Peter Thomas Cartledge, Christian Umuhoza, Natalie McCall

**Affiliations:** 1 Department of Pediatrics, Yale University – Rwanda Human Resources for Health Program, Kigali, Rwanda; 2 Department of Pediatrics, Centre Hospitalier Universitaire de Kigali (CHUK), Kigali, Rwanda; 3 Department of Pediatrics, National University of Rwanda, Kigali, Rwanda

**Keywords:** In-training Examination, Formative Feedback, Medical Education, Global Health, Rwanda, Internship and Residency, Feedback, Perception, Qualitative

## Abstract

**Background:** The University of Rwanda is the only African residency to have implemented the pediatric International In-Training Examination (I-ITE) as a tool to monitor resident knowledge acquisition. The objective of this study was to better understand the acceptance and relevance of this exam to residents from this setting, and their perceptions regarding this assessment tool.

**Methods:** A retrospective, survey-study was undertaken to describe candidate feedback. Immediately on completing the I-ITE residents provided feedback by filling in an electronic questionnaire comprised of four closed Likert questions and an open text box for free-text feedback. Participants were pediatric residents from the University of Rwanda, the only university in Rwanda with a pediatric residency program. Quantitative analysis of the Likert questions was undertaken descriptively using SPSS. Free-text feedback was coded and analysed employing a phenomenological approach, with coding and analysis undertaken by two researchers.

**Results:** Eighty-four residents completed a total of 213 I-ITE sittings during the five exam cycles undertaken during the study period. The survey was completed after 206 of the 213 exam sittings, giving a response rate of 97%. Five themes emerged from the qualitative analysis; 1) undertaking the I-ITE was a positive experience; 2) exam content; 3) formative nature of the assessment; 4) challenges to completing the exam; 5) practicalities to undertaking the exam.

**Conclusion:** Qualitative feedback demonstrates that the I-ITE, a standardized, and independent exam, produced by the American Board of Pediatrics, was valued and well accepted by Rwanda pediatric residents. Its formative nature and the breadth and quality of the questions were reported to positively contribute to the residents' formative development.

## Abbreviations

ABP: American Board of Pediatrics; CBA: computer-based assessment; HRH: Human Resources for Health; I-ITE: International In-Training Examination; IRB: institutional review board; ITE: In-Training Examination; LMICs: low- and middle-income countries; MCQs: multiple-choice questions; PBA: paper-based assessments; PI: Principal Investigator; UR: University of Rwanda; US: United States.

## Introduction

### International In-Training Examination (I-ITE)

For four decades, pediatric residents in the United-States (US) have been given an annual In-Training Examination (ITE) as a formative self-assessment instrument ^1, 2^. The objective of the pediatric ITE is to assess general pediatric knowledge and to track progress year-to-year while being able to compare individual scores with national peers. Research has shown that the ITE examination is a valid and reliable estimate of resident knowledge and can predict final performance in the pediatric board examination ^2– 4^.

The I-ITE is offered annually by the American Board of Pediatrics (ABP) to training institutions globally to assess core areas of general pediatric knowledge and to gauge trainee knowledge acquisition from year to year. The content of the I-ITE is a subset of 150 to 200 multiple-choice questions (MCQs) from the US board certification exam, which is formulated by 30 board-certified experts ^4^. The I-ITE is annually reviewed by physicians (a reviewer and 1-2 medical editors), in an attempt to ensure current/relevant information, and, where possible, to remove any “Americanisms”, terminology, etc. The I-ITE was piloted with one training program in Lebanon in 2008, and made available to all countries in 2009 ^5^. The I-ITE has been shown to be a tool that can measure resident knowledge acquisition and institutional factors supporting residents in a resource-limited country, but has not be studied otherwise.

### Pediatric residency in Rwanda

In 2013, 23 African countries were offering a postgraduate training program in pediatrics ^6^. In Rwanda, there is a single pediatric residency program graduating approximately five to 18 pediatricians per year. Rwanda is the first sub-Saharan nation to employ the pediatric I-ITE to provide formative feedback to individual residents and to give feedback to the faculty on the overall performance of residents within the residency program ^5^. The I-ITE, however, is a formative assessment based on the American ITE examination, and though adapted it is being utilised in a very different cultural and medical context in terms of both the populations, patients and pathologies encountered, but also in respect to the language skills, learning styles and assessment experience of the residents undertaking the assessment. There is currently no literature regarding the acceptance and relevance of this exam to residents from this setting and their perceptions regarding this assessment tool.

### Objectives

This study sought to describe the experiences, satisfaction and acceptability of this online formative assessment within the specific context of Rwandan residents by identifying themes within the written feedback given by Rwandan residents who had taken the I-ITE between 2013 and 2018.

## Methods

### Ethical statement

Ethical approval was gained from the Institutional Review Board (IRB) of the University of Rwanda (UR). Ref: 202/CMHS_IRB/2019. Participation in the exam and giving feedback was deemed as consent, with this consent process having been reviewed and approved by the UR IRB. No personal resident data was used in the analysis. Names were removed and replaced with unique identifiers. No significant physical, social, emotional, legal and/or financial risks to participants were identified.

### Study design

We undertook a retrospective, survey-study, employing quantitative (Likert questions) and qualitative (free-text) data, to describe candidate feedback. The reporting of the qualitative analysis has been verified in accordance with the COREQ checklist for qualitative studies (see *Reporting guidelines* ^7^) ^8, 9^. We sought to gain a rich and detailed understanding of the experiences, attitudes and perceptions of the residents, and for the results to emerge from the data. Our previous paper has demonstrated that the I-ITE exam is a valuable tool to measure knowledge acquisition in Rwandan residents ^5^, whereas this current paper, using qualitative analysis, gave the opportunity to understand the residents’ experience and the cultural relevance to them as end-users.

### Setting

UR, the only university in Rwanda with a pediatric residency program. Residents are trained at four tertiary level teaching hospitals in Rwanda.

### Context

The I-ITE was implemented into the UR pediatric residency program from 2012 to 2018 by the faculty of UR and the Human Resources for Health (HRH) program ^5, 10, 11^. A full description of the implementation of the I-ITE in Rwanda, and the performance of the residents in this assessment, has been previously described in the literature ^5, 12^.

**Figure 1.**
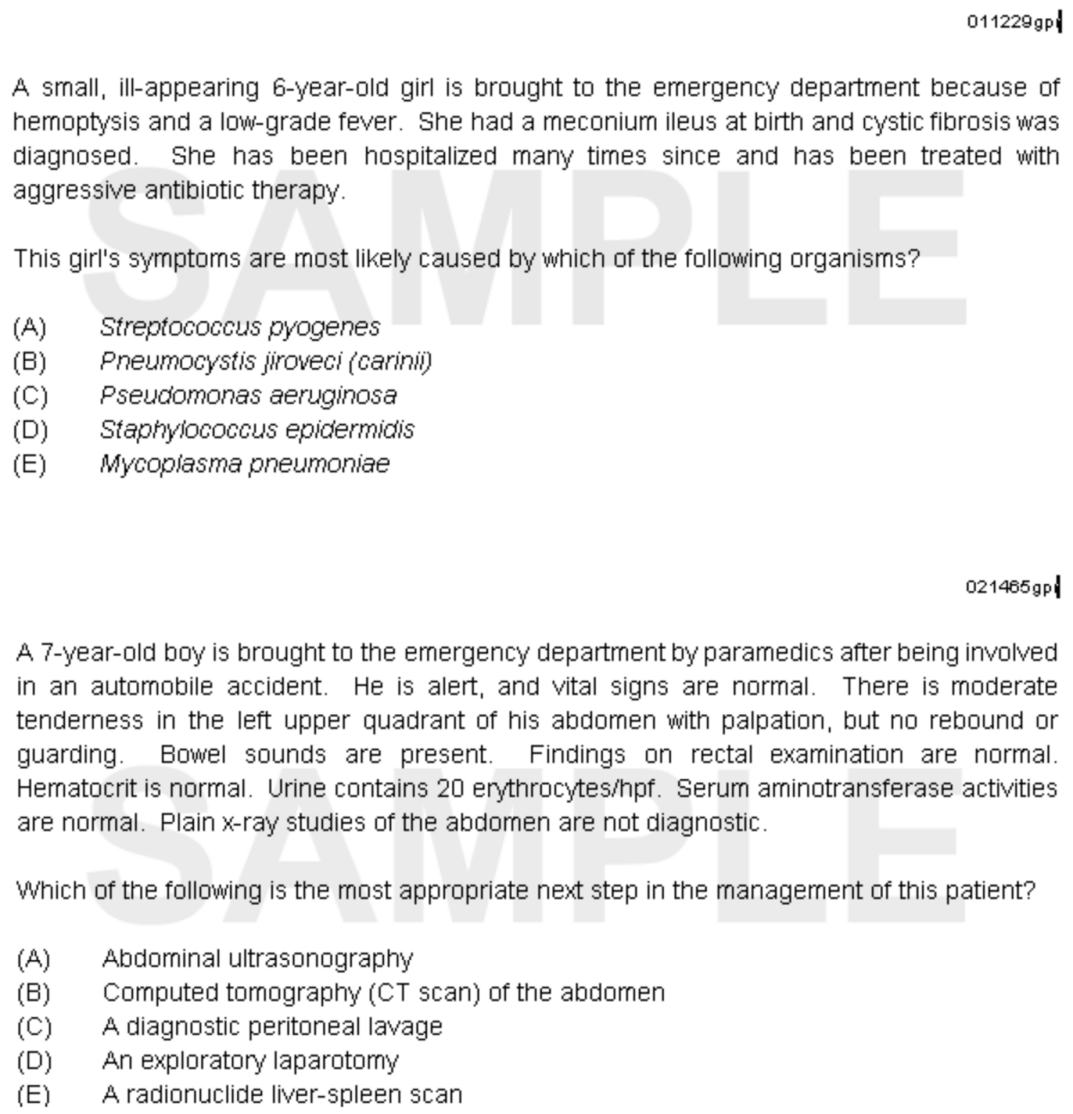
Sample International In-Training Examination multiple-choice questions (provided by the ABP).

### The International In-Training Examination

The I-ITE is an assessment employing single best answer MCQs, executed electronically ( Figure 1), requiring each participant to have access to a computer, usually a laptop, and internet access. The exam is undertaken in a quiet room, proctored by a faculty member.

### Questionnaire, data collection and management

**Figure 2.**
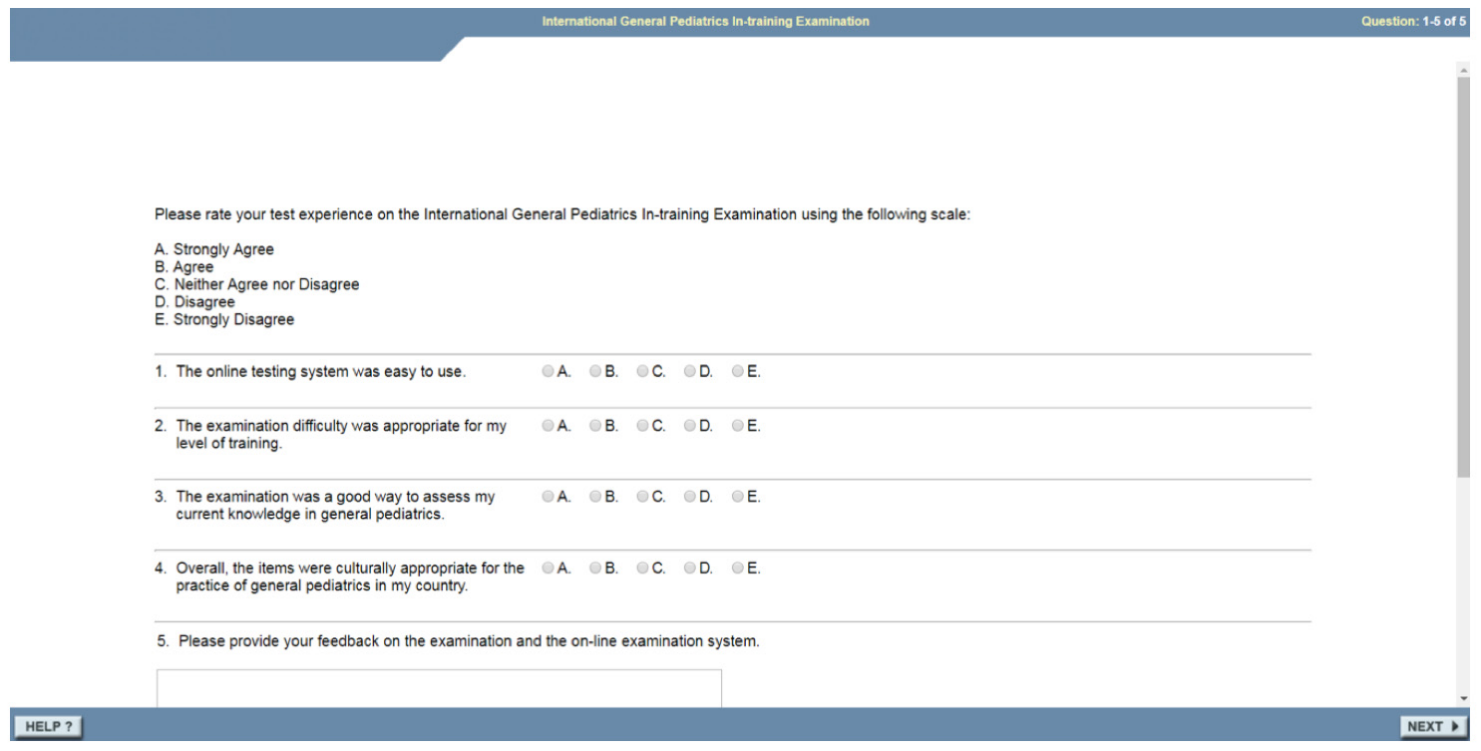
Post-exam questionnaire (provided by the ABP).

On completing the MCQ exam, it is routine ABP practice for residents to provide feedback by filling in a digital/electronic questionnaire comprised of four closed Likert questions and an open text box for free-text feedback ( Figure 2). All questions and feedback were provided in English, the official academic language of Rwanda. The questionnaire was designed by the ABP, and is not a validated tool, therefore the investigators of this study were not involved in the design of the questions used in the feedback as these were standard questions created by the ABP. Completing the Likert questions and free-text comments were not obligatory to complete the I-ITE. The Likert scores and feedback comments were provided by the ABP.

### Participants and sampling

Pediatric residents in Rwanda have completed undergraduate medical school and a minimum of one year as a general practitioner in a district hospital. No sampling methods were used as all resident I-ITE exam sittings were used in the analysis.

### Sample size calculation and saturation

All residents in the program undertook the I-ITE during the period of study (2012–2018), and all data were analyzed; We did not take a sample of this population, as we used the data from the entire cohort, therefore, no sample size calculation was performed, and saturation was not considered as there were no non-participants that could be enrolled or questioned.

### Incentives for participants

The primary objective of implementing the I-ITE was for the formative benefit of the residents. No other incentives were offered to residents to undertake the I-ITE exam or give feedback and participants enrolled in the I-ITE voluntarily.

### Statistical analysis of Likert questions

Quantitative data are reported using descriptive statistics, using Statistical Package for the Social Sciences (SPSS) ^13^.

### Qualitative approach to free-text data

A phenomenological approach was taken. The aim of the free-text analysis was to simply describe the lived experiences of the residents in this setting ^14^.

### Transcription and coding

Free-text comments were submitted electronically by residents immediately after completing each exam sitting; therefore, no transcription was required. Transcripts were not returned to participants for comment or correction. The data was coded by the Principal Investigator (PI, PC), in Microsoft Excel (V16.17.27), with codes identified de novo, as they developed ^15^. Nvivo (V11.4) was used to create a word cloud, combining stemmed words. No codebook was created prior to starting the analysis. Coding was then double-checked by a second investigator (NM) with amendments made.

### Thematic analysis

An inductive approach was taken to analysis. No hypothesis was formed prior to the research. This study employed conventional content analysis. The goal of the content analysis was to “provide knowledge and understanding of the phenomenon under study” ^16^. Themes were not identified in advance. Thematic content analysis was performed by the PI undertaking the following steps ^17^: i) familiarization of the data; ii) identifying codes and themes found in the transcripts by searching for repetition, metaphors, analogies, and “in vivo” categories used by participants; iii) organizing codes and themes for presentation. Participants did not provide feedback on the coding or thematic analysis. The Likert questions allowed for a degree of triangulation of the qualitative data and to assess the trustworthiness of the written, free-text, comments.

### Researcher characteristics and reflexivity

PC is a male, British pediatrician; NM is a female, Swiss-American pediatrician; and CU is a male, Rwandan pediatrician. At the time of the study, PC and NM were working in Rwanda on the Human Resources for Health (HRH) program, and CU is a member of faculty at the University of Rwanda. All investigators are clinicians specializing in pediatric medicine and medical education. The investigators are responsible for supervising UR pediatric resident research activity and collectively have experience undertaking and supervising qualitative research activities.

### Researcher relationships with participants

All three investigators have professional relationships with the residents who were participants in the study. The data was removed of personal identifiable details to minimize bias of the comments made. Though data collection was prospective with feedback provided immediately after the completion of the exam, the analysis of the feedback was retrospective. Therefore, participants were unaware of the personal goals and reasons for doing the research at the time of completing the feedback questionnaire.

### Potential biases

As the feedback was provided digitally and in English this may have biased the feedback to participants who possess better English language skills and those with better IT skills. The data has the potential to be biased by social desirability bias. All three investigators were members of the pediatric residency faculty and therefore, may have held biases and assumptions regarding the I-ITE before undertaking the analysis. No formal steps were taken to measure or alter these biases.

## Results

### Missing data

No data-points were missing in the data provided by the ABP.

### Participants

Eighty-four residents completed a total of 213 I-ITE sittings during the five exam cycles undertaken during the study period. The I-ITE exams were undertaken by residents in November 2012 (n=27), March 2014 (n=41), April 2016 (n=50), March 2017 (n=49) and May 2018 (n=46). Demographic data (age, etc) is not requested in the feedback tool and therefore is not available. The survey was completed after 206 of the 213 exam sittings, giving a response rate of 97%. 

### Quantitative responses to Likert questions

Residents found the online testing system easy to use and felt that the examination was a good formative assessment of their knowledge in general pediatrics ( Table 1). They were more undecided regarding the difficulty of the exam in terms of their level of training. Overall, residents scored that the items were not culturally appropriate for the practice of general pediatrics in Rwanda, though the most common (modal) response of residents were undecided on this question.

**Table 1.** Likert scale quantitative feedback (n=206).

	Mean (SD)	1= Strongly disagree	2= Disagree	3= Neither agree nor disagree	4= Agree	5= Strongly agree
**The examination was a good way to assess my** **current knowledge in general pediatrics.**	**4.10 (±1.0)**	5 (2.4%)	18 (8.7%)	20 (9.7%)	71 (34.5%)	92 (44.7%)
**The online testing system was easy to use.**	**3.81 (±1.1)**	6 (2.9%)	24 (11.7%)	40 (19.4%)	69 (33.5%)	67 (32.5%)
**The examination difficulty was appropriate for** ** my level of training.**	**3.33 (±1.0)**	8 (3.9%)	36 (17.5%)	69 (33.5%)	66 (32%)	27 (13.1%)
**Overall, the items were culturally appropriate for** **the practice of general pediatrics in my country.**	**2.69 (±1.1)**	31 (15%)	60 (29.1%)	71 (34.5%)	29 (14.1%)	15 (7.3%)

### Thematic analysis

**Figure 3.**
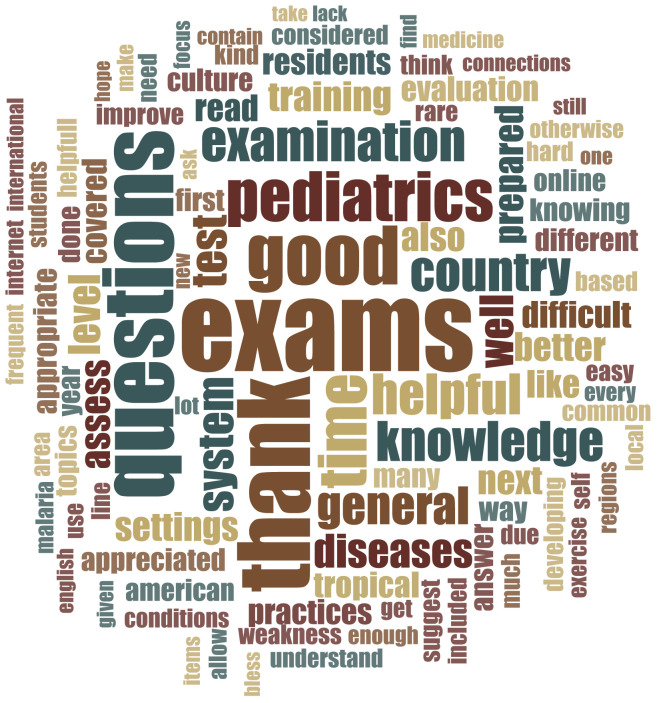
Word cloud of feedback.

Meaningful free-text feedback was given in 160 of the 206 completed surveys (78%). All the comments were made in English. Median comment length was 21 words long (min=2, max=86). Five themes emerged from the analysis ( Table 2). Nvivo was used to produce a word cloud to visualise the commonly used words in the participant feedback ( Figure 3). The sex of the participant and the year of the exam sitting are provided with all the quotes below.

**Table 2.** Thematic tree.

	Theme description	Sub-themes
**Theme 1**	I-ITE as a positive experience	
**Theme 2**	Exam content	Pathology from developed nations
		Pathology from resource-limited nations
		Breadth of the pediatric curriculum
		Item difficulty
		High-quality assessment process
**Theme 3**	Formative nature of the exam	Self-development (formative nature)
		Level of training
		Feedback on answers
**Theme 4**	Challenges to completing the exam	Language challenges
		Time to complete questions
		Internet connection
**Theme 5**	Practicalities	Preparation
		Exam software
		Timing of taking the exam within the academic year
		Desire to undertake I-ITE more frequently

***Theme 1: I-ITE as a positive experience***

Residents consistently reported that undertaking the I-ITE was a positive experience. Codes within this theme were the most commonly coded items during the analysis. Residents valued the experience, the quality of the assessment, and reported that it was suited to their level of training,

“This was a good experience” [F, 2015],
“This exam was incredible. I really appreciated how it was set” [M, 2013]
“This was a really good assesment for my level of training”[M, 2016].

This high level of end-user satisfaction is an important aspect of formative assessments to ensure that residents remain engaged in the process. This is especially important where the I-ITE is offered annually during a four-year program.

***Theme 2: Exam content***

*Subtheme: Pathology from developed nations*

Forty-nine residents commented that the questions tested unfamiliar pathologies, investigations, and terminologies.

“… sit and ask yourselves if knowing American stuff like - Little league - Gun induced shot etc. These are for Americans not for Africans. For us, we deal with malaria, diarrhea, pneumonia and other tropical illnesses” [M, 2017].

The MCQ questions used in the I-ITE are not explicitly written for settings such as Rwanda. This theme was supported by the Likert questions, suggesting that residents did not feel that the questions were culturally or epidemiologically appropriate.

*Subtheme: Pathology from resource-limited nations*

Similar to the presence of American pathology was the lack of pathology relevant to settings such as Rwanda.

“I could suggest that next time you consider also tropical medicine where malnutrition, malaria,.... are a big concern” [F, 2016]

This is an important point worth considering. However, the I-ITE is undertaken in many settings outside of the USA and is not exclusively for the use of resource-limited countries with similar pathologies to Rwanda. Therefore, tailoring it to each country is like to be beyond the scope of the ABP. In addition, the I-ITE is designed to match the ITE given in the US closely. Removing too many questions may modify the ITE to the point of being completely different and, therefore, uncomparable. These comparisons give significant meaning to the formative nature of the assessment process.

*Subtheme: Breadth of the pediatric curriculum*

Formative assessments are important to allow residents to assess their own performance in relation to that expected within a curriculum ^18^. The Rwandan pediatric residency follows a curriculum based upon a modified, locally adapted version of the Global Pediatric Education Consortium curriculum, also developed by the ABP ^19^. Residents reported that the exam was good at assessing the breadth of the curriculum topics of general pediatrics. This was despite the use of Americanisms and pathologies and the lack of locally relevant pathologies.

“This [I-ITE] is fundamental as it provides the base line common knowledge in pediatrics” [M, 2012].

The I-ITE is a formative assessment, and as such, it gives residents a time-specific assessment of their general level of knowledge without being overly sub-specialized. Though not all topics are assessed, it provides residents with benchmarking of the areas for improvement.

*Subtheme: Item difficulty*

A subset of residents found that the questions in the exam were difficult. Reasons for this difficulty were; lack of time, language skills, the relevance of pathology, not having encountered investigations in their setting, length of text in the scenarios, being assessed early in residency, breadth of topics assessed, and the perceived level of knowledge of Rwandan residents.

“this was difficult for our knowledge” [F, 2013]
“many question were not appropriate for the level of my training and also many of them should be differently answered when practicing in my settings” [F, 2015]

The I-ITE scores gained by Rwandan residents were significantly below residents from other international settings ^5^. The difficulty of questions was based on multiple factors. The comments found within this subtheme did not triangulate with the Likert question responses regarding difficulty where residents did not feel that the difficulty was excessive for their level of training. Therefore these free-text comments may have reflected the experiences of a subset of the cohort.

*Subtheme: High-quality assessment process*

The residents reported that the assessment was of a high quality. They reported that the questions were clear and concise and that it was well structured. No residents gave any concerns regarding quality, only the relevance of questions. Comments regarding the assessment being “well prepared” may reflect the involvement of local faculty to ensure that the exam ran smoothly, rather then the I-ITE exams themselves.

“The exam was well prepared and the questions were clear and concise.” [M, 2015, P47]
“The exam was well structured” [M, 2017, P36]
“Good and well prepared examen with easy online system.” [M, 2015, P46]

The I-ITE has undergone rigorous processes to ensure that the quality, structure and format of questions is high, the residents well appreciated this.

***Theme 3: Formative nature of the exam***

*Subtheme: Self-development (formative nature)*

Residents valued the formative nature of the I-ITE exam. Residents felt it helped them to identify areas for personal reading and development. They also valued being able to assess their own level of performance compared to their peers. Many residents also reported that the exam acted as a motivator for future study.

“Thank you for helping me to asssess my level. I discovered my weakness and I am going to improve my area of weaknesses” [M, 2016]
“The examination was hard. But it reminds me to work hard” [M, 2015]

Many residents had a clear understanding of the formative nature of the exam, benchmarking them against peers and identifying areas for improvement. The qualitative responses within this sub-theme were supported by the quantative responses to the Likert question, with nearly 80% agreeing that the exam was good for assessing their level of performance.

*Subtheme: Level of training*

In the US, the pediatric ITE is used by all residents, of all levels, to benchmark themselves, throughout residency, in preparation for their pediatric board exams. Board equivalent exams are not undertaken in Rwanda. Though the feedback (above) demonstrated a degree of general understanding regarding formative assessment, it was evident that many residents had not fully grasped the concept of this formative assessment being employed as a tool to track their own progress over a series of years. For example, several residents wanted bespoke exams based on their level of training at the time of taking the exam, which would not enable a tracking of performance during residency.

“It can be better if the exam is different depending on which level of the training you are” [M, 2015]

There was, however, a small sub-set of first-year residents who appreciated being able to assess their level of knowledge at this early stage in their residency.

“Thank you for the examination, I am resident in first year and it helped me to know my level of weakeness so that I will improve” [F, 2016]

*Subtheme: Feedback on answers*

There was a desire from residents to be given the answers, along with feedback, to the individual question items. This reflects the desire of residents to use the I-ITE as a tool for learning rather then an assessment of their performance.

“It would be better if we get the answers to review them and improve our medical knowledge” [F, 2017]

Undertaking an exam can be stressful, therefore gaining specific feedback on the questions would be helpful to residents.

***Theme 4: Challenges to completing the exam***

*Subtheme: Language challenges*

Kinyarwanda is the official, unifying language of the 12 million population of Rwanda. French, which is still an official language in Rwanda, was used in education in Rwanda until 2010. Many Rwandan residents would therefore have undertaken their primary and secondary education in French and Kinyarwanda. Language was, therefore, a challenge reported by several residents undertaking the I-ITE.

“The exam contain difficult words to understand when you are not english native speaker, which make it more dificult” [F, 2016]

Despite this, many residents reported that the questions were easy to read, understand and were concise.

“The exam is well prepared and easy to read and understand” [M, 2017]

*Subtheme: Time to complete questions*

Residents found the experience of undertaking the I-ITE positive. However, the timing was a common challenge, with residents reporting not enough time to complete the questions.

“The number of questions and length of statement/clinical scenario given with questions do not allow time for reasoning before chosing one most likely answer” [M, 2015]

*Subtheme: Internet connection*

Internet connection for residents in Rwanda is expensive, frequently not available or of low bandwidth. Internet connection was frequently described in the feedback, with many residents reporting internet connection problems during the exam.

“This examination is helpful for our training in general pediatrics because it shows us our level of knowledge internationaly but it is difficult to use online system due to our slow internet connection in our setting” [F, 2017]

***Theme 5: Practicalities***

*Subtheme: Preparation*

Residents had a desire to have sample questions with which to prepare for the I-ITE exam. They also felt that they needed more practice at MCQ format questions.

“I would like to have my scoring rate and sample questions in my mail box in order to prepare the next exam.” [M, 2012]

Residents in Rwanda do not commonly have access to online exam preparation materials, which are often unaffordable or not available due to internet bandwidth.

*Subtheme: Exam software*

The online system/software used for the I-ITE were frequently discussed in the feedback. Comments were almost exclusively positive, with residents finding the system of good quality, user-friendly, and easy to use.

“The online system is user friendly! The exam contained some culrurally irrelevant questions” [M, 2016]

The positive nature reported by residents was corroborated by the high Likert scores reporting that the system was “easy to use”

*Subtheme: Timing of taking the exam within the academic year*

The timing of the exam during the academic calendar is important, with the recommendation being that it comes early in the academic year, in order to have a baseline assessment with which to have a maximum impact in the formative process for residents. The Rwandan I-ITE was often executed late in the academic year and was therefore frequently not undertaken at this optimum time due to the practicalities of enrolment and payment.

Interestingly, residents reported conflicting opinions on the timing that they wished to undertake the exam in the academic calendar. Some residents wanted the exam early to give time to prepare for the end of year exam.

“I also suggest that if possible this exam could be taken earlier, in the beginning, or in the middle of the academic year (december-january), as it can helps students to keep reading and prepared” [M, 2017]

Whereas, first-year residents generally found undertaking the exam early in their studies a negative experience.

“… and i hope that the next evaluation will be at the end of the next academic year because this one was very early only 2 months for our study …. ” [M, 2012]

*Subtheme: Desire to undertake I-ITE more frequently*

Residents reported a desire to undertake the I-ITE more frequently in order to be able to assess their level of knowledge. Different participants recommended using the exam; every three months, every trimester, or every six months.

“The exam was a very good way to assess my level of knowledge. I would love to have frequent access to this examination material to train myself. Thank you!” [F, 2015]

In the US, residents undertake the ITE exam annually to gauge their preparation and readiness for the board exams. As residents in Rwanda do not take an equivalent to the board exam, their objectives to take the exam may have been different, and undertaking the exam frequently would allow them to track their performance and identify areas for improvement.

## Discussion

This survey-study sought to describe the experiences, satisfaction and acceptability of this online formative assessment within the specific context of Rwandan residents by identifying themes within the written feedback given by Rwandan residents who had taken the I-ITE between 2013 and 2018.

### Undertaking the I-ITE was a positive experience

Residents valued the I-ITE and reported that it was a positive experience. This was triangulated by their desire to undertake the I-ITE more frequently and their quantitative responses to the Likert questions. The feedback from our residents demonstrates that faculty from other training centers, similar to Rwanda, can confidently implement the I-ITE and that residents will positively receive this formative experience. It is important to note that this feedback was given immediately after the exam and could have been different if the feedback was given after they received their grade score. The formative nature of the I-ITE is to identify residents who may need additional support, and we did not take any steps to evaluate if residents in difficulty find that this aspect of the process causes anxiety or distress and therefore alters their perception of the exam being a positive experience. This would make an interesting piece of qualitative work in the long term. In the short term, faculty implementing the I-ITE should put systems in place to support residents when using the exam scores to identify residents who need additional support in their training.

***Exam content***: Vetting questions for quality and relevancy is critically important when creating both formative and summative assessments ^20, 21^. The residents commented on the relevancy of the questions and topics to the practice of Pediatrics in Africa and commented on the fact that there were questions about diseases and pathology which are not routinely found locally. Conversely, residents also reported that they wish to have seen more questions about pathology more frequently seen locally, such as malaria or malnutrition.

While developing the bank of questions for the I-ITE, the ABP had one or more medical editors to review the I-ITE to ensure current/relevant information and to remove any Americanisms before each year’s administration ^12^. However, because the I-ITE has to be comparable to the ITE undertaken by the residents in the US, only a limited number of questions could be adapted or removed. From the residents' observations, one of our key recommendations is for the ABP to review this process carefully and to choose reviewers who have practiced outside the US to ensure that they include questions that are relevant globally, not only in the US.

***Exam difficulty***: The perceived difficulty of an exam can impact the evaluation of didactic teaching by learners ^22^. More residents felt that the difficulty was appropriate (45%) compared to those who felt it was inappropriate (21%), with the remainder being non-conclusive. This was not reflected in the free-text feedback. The difficulty was reported to be related to: i) topics that were not commonly seen in Rwandan clinical practice, ii) did not correspond to the curriculum taught, and iii) the question format, which included question stems with long case descriptions and significant information to process. Some residents reported that case-based MCQ represented a new style of questions that they previously had not experienced. It is known that student’s satisfaction with MCQ exam questions can correlate with the format of the MCQ and that case-based MCQs, which measure higher reasoning skills (rather than simple memorization or recall of knowledge), can be perceived as being more difficult ^23, 24^. Our own personal experience, in Rwanda, is that official medical school and residency written examinations continue to use a higher proportion of true-false style questioning rather than a true “best-of” approach, and also more factual knowledge assessment rather than case-based reasoning skills. This questioning style could have contributed to this perception of item difficulty in the respondents.

The residents acknowledged the high quality of the tool. For formative assessment to be effective, the tool needs to be credible ^25^. Without credibility, residents would not be able to trust the outcomes of the assessment and therefore, may be less likely to implement any learning strategies from the formative process. It is therefore very reassuring that none of the residents in our cohort expressed concerns regarding the quality of the questions themselves.

### Formative nature of the exam

Our residents found that the formative nature of the exam helped them to identify areas for personal reading and development. Research has demonstrated that the formative nature of the ITE goes beyond knowledge acquisition and improves the resident’s self-assessment skills of their own overall performance ^18^. This is an important finding as the I-ITE not only gives them a single benchmark but provides the skills to self-assess performance and competencies in the future.

Our results suggest that many of our residents did not understand some of the concepts of formative assessment tracking performance over the course of their residency. They reported a desire for an exam that was bespoke to their own level of training (e.g., as a first-year resident). This highlights the importance of preparing residents for the I-ITE to understand that it is a formative process over a number of years and that they can track their own progress and identify where their current performance measures in comparison with their peers and their previous scores. This should be done in a manner to encourage residents to take ownership of their own learning and their own formative assessment ^25^.

When undertaking a formative assessment, there is a natural tendency to want to understand each question and gain feedback that goes beyond a quantitative benchmark. This finding was found in residents preparing for the ITE using question format preparation materials ^26^. However, this kind of feedback would compromise the exam bank and would not be feasible.

***Challenges to completing the exam***. Assessing knowledge in our Rwandan residents who are not native English speakers was problematic. Though the official language of education in Rwanda is English, residents will complete any formative and summative assessments in English. The feedback identified that some residents found that completing the I-ITE in English was challenging. We, therefore, postulate that they would also have similar concerns completing their UR summative exams in English. However, it does also add to the formative nature of the I-ITE as it will have allowed residents to gain feedback on their own language comprehension in preparation for their summative assessments, undertaken in English.

### Implementing the I-ITE in other settings

Residents valued the formative nature of the exam. However, this impact of a formative exam goes beyond user satisfaction. The use of an annual, formative, in-training examination in Pakistani residency programs, in combination with a number of further interventions, significantly increased the number of residents successfully completing their postgraduate examinations ^27^. Therefore, regular formative assessment has the potential to improve long term outcomes for residents and other residency programs in resource-limited settings, such as Rwanda, may benefit from implementing the I-ITE. The benefits are to implement a high-quality formative process without requiring a substantial burden on the faculty. There are several considerations that these programs should consider when implementing the exam. Several residents reported that the amount of time available to complete each question was not sufficient. Being able to undertake questions promptly is a necessary skill when undertaking summative MCQ-style questions and is, therefore, in itself formative for the participants. After the first year of the I-ITE implementation, the amount of time given for the exam was extended. Undertaking an examination can be a stressful experience for participants; therefore, identifying methods to overcome practical challenges, such as internet connection, would likely lead to a more positive experience and more engagement with the process ^28^.

Though residents themselves did not discuss it, the I-ITE is not only formative for individual residents, but it also offers a high-quality predictor of residents in difficulty who may need early intervention to ensure they meet the required knowledge and competencies ^29^. One residency, in the USA, implemented interventions for low scoring residents including: closer academic guidance and supervised meetings with a faculty advisor, development of a personalized study plan with an emphasis on self-study and reading, meeting with a test-taking expert, mandatory departmental didactic conference attendance and withdrawal of moonlighting privileges ^30^. The combination of these steps was highly effective at improving resident performance. Therefore, faculties considering implementing the I-ITE should predefine the objectives of doing so, and how the strategies they will use to support residents who are underperforming.

### Supporting residents to familiarise themselves with the exam format

Research has shown that residents who prepare for examinations by completing review questions result in quantitatively higher ITE scores ^31^. It is therefore interesting to note that our residents reported a desire for sample questions with which to prepare for the exam format. The ABP, or individual departments implementing the I-ITE, may want to collaborate to create a bank of sample questions to help prepare residents for the I-ITE and other formative and summative assessments. This will give residents familiarity with the style of questions used, rather than comprehensive coverage of the pediatric syllabus. It is important to note, however, that personal study is not necessarily the optimum method for preparing for such exams, with one study finding that residents undertaking a higher number of general pediatric admissions correlated with an increase in ITE score ^32^.

### Practicalities of implementing the exam

It is reported in the literature that computer-based assessments (CBA) are generally well-received for formative assessments ^26, 33^. Establishing the use of a CBA is not straightforward and can be negatively received. Implementation of a CBA requires a series of steps, namely; introducing CBA to both staff and students, using an established item bank, ensuring adequate infrastructure, developing CBA software, piloting the CBA, and making preparations for untoward incidents, and finally evaluating the exam ^34^. The use of the I-ITE from a reputible source allowed for the majority of these steps to be undertaken without involvement of the local faculty, and we feel that this contributed to the positive satisfaction amongst our residents.

### Transferability

Our residents have their own perceptions regarding assessment processes, which are formed within their own environment and culture. Therefore, though several important themes have been drawn from their feedback, these may not be fully transferrable to other resource-limited settings who are considering implementing the I-ITE.

### Future research work

Future pieces of research work may be to assess local faculty engagement and perceptions of the I-ITE qualitatively. Participants had commented on the "Americanisms" in the I-ITE, and many undertook the exam in their “non-native” language. Therefore, if the I-ITE applied in other settings with a larger cohort of residents, a very interesting piece of prospective work would be to undertake English language assessments and compare this with candidates I-ITE outcomes.

### Study limitations

The single major limiting factor of the study was that the Likert and free text questions were not explicitly designed for the purpose of this study and rather designed by the ABP for all residency programs. Interviews or focus-groups could, therefore, have provided more rich data and a more comprehensive understanding of the residents' perspectives and experiences. Due to the qualitative nature of the methodology, we can only discuss what the participants reported and not what they do or did. The personal experiences and pre-existing opinions of the researchers may have biased the interpretation of the qualitative data. Just as some residents may have found it difficult to complete the I-ITE in English, these same residents may have found giving feedback in English challenging.

## Conclusion

We demonstrate that the I-ITE, a standardized, and independent exam, produced by the ABP, is valued and well accepted by pediatric residents in Rwanda. Its formative nature and the breadth and quality of the questions were reported to contribute to the residents formative development positively. Residents from resource-limited settings with limited faculty and didactic teaching wish to have more frequent access to formative learning opportunities such as this type of assessment. Adapting the exam to make it more relevant to local epidemiology would be beneficial to the end-user, in their own setting and allow for a better comparison of knowledge between settings. The ABP should be applauded for producing this resource for international residencies. The low number of residencies using the I-ITE in low-income countries (LICs) should also be a point for reflection. The cost of purchasing the I-ITE is probably beyond the reach of many institutions and was only made possible in Rwanda because of the HRH programme and externally funded programme. Therefore, the ABP may wish to consider taking a HINARI approach and offer the I-ITE free of charge in LICs as a gesture of their desire to see pediatric training and practice improve in these settings.

## Data Availability

The datasets generated and/or analyzed during the current study are not publicly available as they are third party data owned by ABP. They can potentially be made available from the corresponding author on reasonable request for the purpose of further research. This would require providing a research protocol and consultation with the American Board of Pediatrics (data owners) and the authorizing Institutional Review Board (University of Rwanda). Harvard Dataverse: COREQ checklist for “Experiences of the International In-Training Examination (I-ITE) by Rwandan pediatric residents – a mixed-methods description of candidate feedback”. https://doi.org/10.7910/DVN/5X5XWT ^7^. Data are available under the terms of the Creative Commons Zero "No rights reserved" data waiver (CC0 1.0 Public domain dedication).
