# Supplementary material for: Experiences of the International In-Training Examination (I-ITE) by Rwandan pediatric residents – a survey-study describing candidate feedback
Source: F1000Res. 2023 Jan 6;9:1448. Originally published 2020 Dec 11. [Version 2] doi: 10.12688/f1000research.27293.2 (PMC11809634; doi:10.12688/f1000research.27293.2)
Supplement: Peer review history

# Reviewer report: Reviewer response for version 2

**DOI:** 10.5256/f1000research.142445.r223733

**Published:** 2025-01-09

**Authors:** Mantoa Mokhachane, Firdose Nakwa

**Affiliations:** 1 Wits University, Johannesburg, South Africa; 2 University of the Witwatersrand Johannesburg Faculty of Health Sciences, University of the Witwatersrand Johannesburg Faculty of Health Sciences, Johannesburg, Gauteng, South Africa

Review by Professor Mantoa Mokhachane (mantoa.mokhachane@wits.ac.za) and Professor Firdose Nakwa (Firdose.Nakwa@wits.ac.za).

**Summary**

 This is a retrospective survey aiming to understand the acceptance and relevance of the pediatric International In-Training Examination (I-ITE) as a tool to monitor resident knowledge acquisition and their perceptions regarding this assessment tool. ‘Quantitative analysis of the Likert questions was undertaken descriptively using SPSS. Free-text feedback was coded and analysed employing a phenomenological approach, with coding and analysis undertaken by two researchers.’

**Comments**

General

 • We were curious to know how a foreign assessment could work in an African context.

Abstract

 • Wondering whether the first sentence could phrased differently from “The University of Rwanda is the only African residency to have implemented the pediatric International In-Training Examination (I-ITE) as a tool to monitor resident knowledge acquisition”

 To ….is the African country to have implemented the pediatric International In-training Examination (I-ITE) as a tool to monitor resident knowledge acquisition in its residency programme.

 • Was relevance or context to the African situation not a theme that came out?.

Methods

 • Researchers employed phenomenology to code free text

 o Which type of coding did they use – open coding, axial or selective coding?

 • Which language was used to conduct the exam? This is important as it could have influenced the outcome of the results.

 • Was I-ITE part of the annual assessment? Was a compulsory or was a possibility of opting out?

 • How long is the residency programme and do residents take the same exam every year?

 • The authors mention that the test is taken early – is it only in the first year of residency or annually?

Ethics

 Did the researchers request permission from the residents to use this data in the future? If not, are they now asking for permission prospectively?

Results

 • Why can’t the local pediatric board remove the American content and replace with culturally appropriate and contextual content? Is this possible going forward?

 • When and how often do the Americans take this exam? Is it compulsory?

 • When is the academic year?

Is the work clearly and accurately presented and does it cite the current literature?

Yes

If applicable, is the statistical analysis and its interpretation appropriate?

Partly

Are all the source data underlying the results available to ensure full reproducibility?

No

Is the study design appropriate and is the work technically sound?

Partly

Are the conclusions drawn adequately supported by the results?

Partly

Are sufficient details of methods and analysis provided to allow replication by others?

Yes

Reviewer Expertise:

Neonatology, Paediatrics, Medical Education and Ethics

We confirm that we have read this submission and believe that we have an appropriate level of expertise to confirm that it is of an acceptable scientific standard.

# Reviewer report: Reviewer response for version 1

**DOI:** 10.5256/f1000research.30158.r138352

**Published:** 2022-05-24

**Authors:** Gary L Beck Dallaghan

**Affiliations:** 1 Office of Medical Education, University of North Carolina at Chapel Hill School of Medicine, Chapel Hill, North Carolina, USA

I was interested to read this article to see how an American examination would translate for use in a culturally diverse part of the world. I appreciate the authors' work on this study. The following comments are intended to help them make revisions to this study.

 I'm starting with the abstract first primarily because it is the first thing one reads. After reading the entirety of the manuscript, I would argue that you did not do a mixed methods study but rather a survey study that included both quantitative and qualitative sources of data. Mixed methods requires much more deliberate planning to ensure that both quantitative and qualitative data instruments complement one another. What you did was use an established tool designed by the American Board of Pediatrics that was intended more as a feedback loop. It does not meet the criteria to classified as mixed methods. You may disagree, but I would seriously suggest you consider this.

 Because of that, if this is intended to be survey research, you only need one response rate. That portion of your abstract was confusing to read because it is not the norm to report a response for the quantitative vs qualitative data. Since this data was submitted simultaneously, you have one response rate.

 Introduction section read fine to me. It was very clear.

 Methods: Again, rethink this notion of mixed methods.

 In the qualitative approach you indicate you did not use a guiding theory. However, in the thematic analysis section you reference describing the participants' lived experience. That is known as phenomenology, which is a guiding theory. I would recommend reading a bit about phenomenology and decide if that is what you should have stated. These are contradictory statements that need to be resolved.

 Results and Discussion read well to me. I did appreciate you triangulating quantitative findings with your themes.

 It was quite interesting to read the comments from people who noted the "Americanisms" in the examination and how challenging that was. I also wonder if there is room for future research related to how people do based on their English proficiency.

Is the work clearly and accurately presented and does it cite the current literature?

Yes

If applicable, is the statistical analysis and its interpretation appropriate?

Partly

Are all the source data underlying the results available to ensure full reproducibility?

No

Is the study design appropriate and is the work technically sound?

Partly

Are the conclusions drawn adequately supported by the results?

Yes

Are sufficient details of methods and analysis provided to allow replication by others?

Yes

Reviewer Expertise:

I am an educational psychologist with extensive quantitative and qualitative research experience.

I confirm that I have read this submission and believe that I have an appropriate level of expertise to confirm that it is of an acceptable scientific standard, however I have significant reservations, as outlined above.

# Response

**Published:** 2022-12-20

**Authors:** Peter Cartledge

**Affiliations:** Yale University (USA) Human Resources for Health (Rwanda), Rwanda

We would like to thank you for your helpful comments, and review. We have amended the article based on this feedback. Responding specifically to each comment:

**Comment: **I'm starting with the abstract first primarily because it is the first thing one reads. After reading the entirety of the manuscript, I would argue that you did not do a mixed methods study but rather a survey study that included both quantitative and qualitative sources of data. Mixed methods requires much more deliberate planning to ensure that both quantitative and qualitative data instruments complement one another. What you did was use an established tool designed by the American Board of Pediatrics that was intended more as a feedback loop. It does not meet the criteria to classified as mixed methods. You may disagree, but I would seriously suggest you consider this.

**-Response: **Yes, this is very reasonable and appropriate. We have changed the wording in the methods and the abstract to reflect this. We have also changed the title to reflect this.

**Comment: **Because of that, if this is intended to be survey research, you only need one response rate. That portion of your abstract was confusing to read because it is not the norm to report a response for the quantitative vs qualitative data. Since this data was submitted simultaneously, you have one response rate.

**-Response:** Thank you for this, and certainly changing it to make it more readable will be helpful. We’ve put a single response rate in the abstract. We’ve moved the response rate in the methodology into the participants section, to reflect a single response rate. Also, we have changed the section in the free-text (qualitative) section to represent the 160 of the 206 responders. We have also changed it in the abstract.

**Comment: **Methods: Again, rethink this notion of mixed methods.

**-Response:** As above. We have reworded this.

**Comment: **In the qualitative approach you indicate you did not use a guiding theory. However, in the thematic analysis section you reference describing the participants' lived experience. That is known as phenomenology, which is a guiding theory. I would recommend reading a bit about phenomenology and decide if that is what you should have stated. These are contradictory statements that need to be resolved.

**-Response: **Thank you, this is really helpful. As you will see from our wording regarding “lived experienced” this was something that we ourselves had difficulty fully deciding on during the design and write-up phase. Having your insight on this is helpful and we have changed the wording. We have also added the reference of “Creswell J. Chapter 4: Five Qualitative Approaches to Inquiry. In: Qualitative inquiry and research design: Choosing among five approaches. 2007. p. 53–84.” which we have used during the process.

**Comment: **It was quite interesting to read the comments from people who noted the "Americanisms" in the examination and how challenging that was. I also wonder if there is room for future research related to how people do based on their English proficiency.

**-Response: **Yes, this is really interesting and one that we frequently reflected on. Rwanda has gone through a significant transition of language skills and moving forward this issue would be significantly less of a problem as many of the newer residents aren’t francophone educated at secondary school. However, if the I-ITE was used in other settings, especially if a larger cohort, then this would make a really interesting piece of comparative research. We’ve expanded on this in the “future research work” section of the manuscript.

# Reviewer report: Reviewer response for version 1

**DOI:** 10.5256/f1000research.30158.r80914

**Published:** 2021-03-19

**Authors:** Ali Dabbagh

**Affiliations:** 1 Cardiac Anesthesiology Department, Anesthesiology Research Center, School of Medicine, Shahid Beheshti University of Medical Sciences, Tehran, Iran

1. First of all, the authors should describe fully how they created their questionnaire; was the validity and reliability of the questionnaire measured? 
2. Why the authors preferred to select no sample size calculation?
3. In the results, did they perform factor analysis?
4. In the discussion, is it possible to describe the results based on each level of the residents?
5. Finally, are the quantitative results described in full? Where?

Is the work clearly and accurately presented and does it cite the current literature?

Yes

If applicable, is the statistical analysis and its interpretation appropriate?

Partly

Are all the source data underlying the results available to ensure full reproducibility?

Yes

Is the study design appropriate and is the work technically sound?

Partly

Are the conclusions drawn adequately supported by the results?

Yes

Are sufficient details of methods and analysis provided to allow replication by others?

Partly

Reviewer Expertise:

Anesthesiology, medical education

I confirm that I have read this submission and believe that I have an appropriate level of expertise to confirm that it is of an acceptable scientific standard, however I have significant reservations, as outlined above.

# Response

**Published:** 2022-12-20

**Authors:** Peter Cartledge

**Affiliations:** Yale University (USA) Human Resources for Health (Rwanda), Rwanda

We would like to thank you for your helpful comments, and review. We have amended the article based on this feedback.

 First of all, the authors should describe fully how they created their questionnaire; was the validity and reliability of the questionnaire measured?

**-Response: **Thank you for your feedback and comments. The questionnaire was designed by the American Board of Paediatrics. In the original manuscript, we had the text “the investigators of this study were not involved in the design of the questions used in the feedback questionnaire as these were standard questions used by the ABP.” In the revised text we have added “The questionnaire was designed by the ABP, and is not a validated tool, therefore…” prior to this to make it more clear. We have have also moved the general text about the I-ITE in general into a new subheading called “The I-ITE”.

 Why the authors preferred to select no sample size calculation?

**-Response: **We did not take a sample of this population, as we used the entire cohort. As no sample was used (i.e. 100%/the full cohort) we didn’t undertake a sample size calculation, and saturation was not applicable as there were no further persons we could interview or enquire of. We have added some extra text to this section to make it a bit more clear.

 In the results, did they perform factor analysis?

**-Response:** We did not undertake any factor analysis. Our data is presented in a descriptive and pragmatic approach; therefore we do not wish to over-reach what is a relatively simple descriptive analysis.

 In the discussion, is it possible to describe the results based on each level of the residents?

**-Response: **We did not do this as it was not described within the objectives of the study. The reasons for this was that the questionnaires were implemented over multiple years and therefore there would be a lot of confounders that would make any analysis / interpretation difficult to undertake.

 Finally, are the quantitative results described in full? Where?

**-Response: **The quantitative results are described in full in Table 1. We used these Likert question responses to triangulate the free text comments.
